# Professor Aquira Jibha ‘Jabu’ Mbokazi, 27 July 1952-02 May 2026: A life dedicated to Family Medicine and service

**DOI:** 10.4102/safp.v68i1.6395

**Published:** 2026-07-30

**Authors:** Olukayode Ademola Adeleke

**Affiliations:** 1 Department of Family Medicine and Rural Health, Faculty of Medicine and Health Sciences, Walter Sisulu University, Mthatha, South Africa

## Early life and education

Professor Aquira Jibha Mbokazi, affectionately known as Jabu, was born in Louwsburg, KwaZulu-Natal, on 27 July 1952. He was the eighth of 11 children born to Reverend Amos and Christina Mbokazi and was raised in a Christian family that valued education, service and faith. His father, a pastor, frequently moved with the family between communities and was committed to ensuring access to education and healthcare. Wherever he was posted, he worked with local communities to establish schools and clinics where these services did not exist. A clinic built through these efforts still stands next to the Mbokazi family home today. These early experiences shaped Jabu’s lifelong commitment to improving healthcare, strengthening communities and developing future generations of healthcare professionals.

He obtained a Bachelor of Science degree in Biochemistry and Chemistry from the University of Zululand in 1975 and Bachelor of Medicine and Bachelor of Surgery from the University of Natal in 1981. He later obtained a Master of Medicine in Family Medicine, a FCFP (SA) and several other postgraduate qualifications.

## Leadership in Family Medicine and health professions education

Professor Mbokazi began his career in private practice in uMlazi while also serving as a district surgeon, preceptor and lecturer in the Department of Family Medicine at the University of Natal. After more than two decades, he joined the Medical University of South Africa (MEDUNSA), where he led the Family Medicine satellite department in Polokwane and later served as Chief Specialist of Family Medicine and Director of the Medical School. His work in Polokwane reflected the values that shaped his upbringing: a commitment to community development, equitable access to healthcare, and a belief in the transformative power of education.

As an internationally respected leader, he fostered collaborations with government, institutions and international partners to advance Family Medicine education and global health initiatives. He was appointed Adjunct Assistant Professor of Family Medicine and Global Health at Des Moines University in Iowa, United States.

In 2018, he became Dean of the Faculty of Health Sciences at Walter Sisulu University, where he strengthened teaching, research, service and community engagement. He maintained the accreditation of key programmes and championed socially accountable health professions education.

Professor Mbokazi provided leadership in professional regulation and research ethics. He chaired the Medical Education, Training and Registration Committee of the Health Professions Council of South Africa and served on several provincial and national health committees. At the time of his passing, he was a member of the National Health Research Ethics Council (NHREC), appointed by the Minister of Health, and served as Deputy Chair of the Committee for Queries and Complaints.

## Legacy

An accomplished family physician, teacher, researcher and mentor, Professor Mbokazi dedicated his career to strengthening Family Medicine, advancing equitable health professions education and nurturing future generations of healthcare professionals.

He is survived by his wife, Ntombikayise ‘Khubi’ Mbokazi, his four children, five grandchildren and four siblings. His contributions will continue to shape the practice of Family Medicine, Primary Health Care, and Health Professions education in South Africa.

**FIGURE 1:**
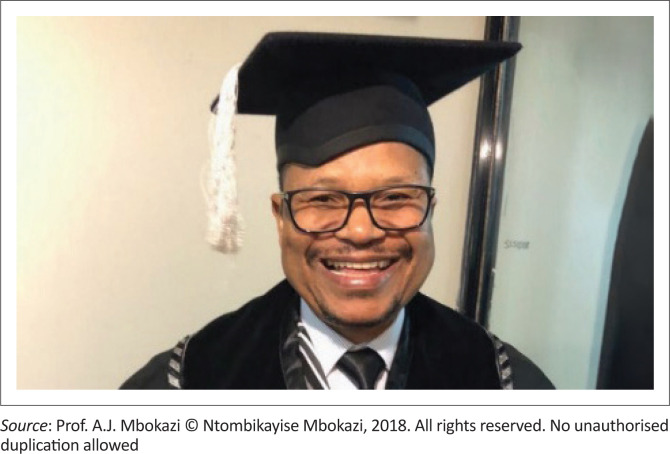
Prof. A.J. Mbokazi at Walter Sisulu University graduation in Mthatha, Eastern Cape on 20 September 2018.

